# Identifying individuals at-risk of developing Parkinson’s disease using a population-based recruitment strategy: The Healthy Brain Ageing Kassel Study

**DOI:** 10.1038/s41531-025-01008-w

**Published:** 2025-07-18

**Authors:** Sebastian Schade, Soumyabrata Ghosh, Alicia Garrido, Philipp Mahlknecht, Tainá M. Marques, Corinne G. C. Horlings, Sonja R. Jónsdóttir, Elisabeth Lang, Claire Pauly, Kavita Rege, Susana Schnell, Maritta Starke, Horst Hakelberg, Eduardo Tolosa, Claudia Trenkwalder, Tamara Wicke, Healthy Brain Ageing (HeBA) Consortium, Sebastian Schade, Soumyabrata Ghosh, Alicia Garrido, Philipp Mahlknecht, Tainá M. Marques, Corinne G. C. Horlings, Sonja R. Jónsdóttir, Elisabeth Lang, Claire Pauly, Kavita Rege, Susana Schnell, Maritta Starke, Horst Hakelberg, Eduardo Tolosa, Claudia Trenkwalder, Tamara Wicke, Gregor Bletzacher, Atbin Djamshidian, Hannah Egger, Iris Egner, Fernanda Farfan, Tobias Fischnaller, Lena Gatterer, Valentin Groues, Somaye Hajian-Tilaki, Beatrice Heim, Sahra Henze, Florian Krismer, Simon Leiter, Kathrin Marini, Deborah Mcintyre, Ulf Nehrbass, Alastair Noyce, Clarissa P. C. Gomes, Noelia Peña Arauzo, Daniel F. Pilco-Janeta, Rajesh Rawal, Susanne Scaglione, Sabine Schmitz, Reinhard Schneider, Anette Schrag, Katharìna Schwarzovà, Klaus Seppi, Verena Seppi, Raquel Severino, Cristina Simonet, Ruxandra Soare, Heike Stockner, Christoph Theyer, Elodie Thiry, Olena Tsurkalenko, Ludmilla Vasilev, Carlos Vega, Liliana Vilas Boas, Laura Zamarian, Rejko Krüger, Maria J. Marti, Werner Poewe, Venkata P. Satagopam, Brit Mollenhauer, Rejko Krüger, Maria J. Marti, Werner Poewe, Venkata P. Satagopam, Brit Mollenhauer

**Affiliations:** 1 Paracelsus-Elena-Klinik Kassel, Kassel, Germany; 2 Luxembourg Centre for Systems Biomedicine (LCSB), University of Luxembourg, Esch-sur-Alzette, Luxembourg; 3 Parkinson’s disease & Movement Disorders Unit, Neurology Service, Hospital Clínic de Barcelona, Institut d’Investigacions Biomèdiques August Pi i Sunyer (IDIBAPS), University of Barcelona and Centro de Investigación Biomédica en Red sobre Enfermedades Neurodegenerativas (CIBERNED: CB06/05/0018-ISCIII), Barcelona, Spain; 4 Department of Neurology, Medical University of Innsbruck, Innsbruck, Austria; 5 Luxembourg Institute of Health (LIH), Strassen, Luxembourg; 6 Centre Hospitalier de Luxembourg (CHL), Strassen, Luxembourg; 7 Department of Neurosurgery, University Medical Center Goettingen, Goettingen, Germany; 8 Department of Neurology, University Medical Center Goettingen, Goettingen, Germany; 9 Queen Mary University of London, London, United Kingdom; 10 University College London, London, United Kingdom

**Keywords:** Diagnostic markers, Parkinson's disease, Predictive markers, Risk factors, Signs and symptoms

## Abstract

Neurodegeneration in Parkinson’s disease (PD) occurs before motor features develop: assessing risk factors and identifying prodromal markers is necessary to recruit prodromal cohorts, improve early diagnosis, and develop preventive therapies. As part of Healthy Brain Ageing (HeBA), we implemented a stepwise, population-based screening to identify people at high risk of developing PD. Residents (*n* = 158,818; 50-80 years) in and around Kassel, Germany were invited to complete an online survey with questions for prodromal symptoms and risk factors. An individual risk score was calculated (International Parkinson and Movement Disorder Society criteria). Selected individuals received a smell test. 8001 of 8774 survey responses were valid; the response rate to the smell test mailings (*n* = 3021) was 90%. Hyposmic participants (*n* = 1019) had more subjective hyposmia and subjective memory impairment (*p* < 0.01). Follow-up visits will validate the recruitment strategy and monitor conversion to manifest PD. Our recruitment strategy identifies people who might be at risk for PD.

## Introduction

The global population of people over 65 years is set to rise rapidly in the next few decades. This rise will inevitably be accompanied by an increase in the prevalence of Parkinson’s disease (PD)^1^, a disorder characterized by progressive α-synuclein (aSyn) aggregation and dopaminergic neuronal loss for which no disease-modifying treatment is available. One reason for the failure of putative neuroprotective strategies is that by the time PD is diagnosed through motor symptoms, a majority of dopaminergic neurons of the substantia nigra is already degenerated^2,3^ and the disease is far too advanced to compensate for the progressing dopaminergic deficit.

The so-called “prodromal” or “premotor” phase of the disease is known to last from 15–20 years, or even 30–40 years in some cases. This phase is characterized by non-motor symptoms (NMS), such as hyposmia, depression, and rapid eye movement (REM) sleep behavior disorder (RBD). Identifying people at-risk for manifest PD is critical, not only for understanding how the disease progresses during its early stages, but also because the preclinical period may be the most opportune time to apply therapies aimed at delaying or preventing progression to motor disease^4,5^.

In recent years, studies of several at-risk cohorts have attempted to identify biomarkers that indicate the risk of an aSyn-related disorder at an early stage^6^. Currently established biomarkers include dopamine transporter imaging (DaT SPECT) and aSyn seed amplification assays (SAA) in cerebrospinal fluid (CSF), both of which indicate dopaminergic dysfunction or pathological aSyn aggregation many years before the onset of motor PD. These findings have led to an on-going debate and new proposals for diagnosing and staging PD from a biological perspective^7^.

In the near future, individuals with prodromal PD will be able to participate in platform trials assessing putative neuropreventive therapies that failed in early, de novo (clinically manifest) PD. Strategies like Path-to-Prevention (P2P) aim to treat at-risk individuals with agents developed for PD and are currently being tested in clinical trials or have previously failed in participants in later disease stages^8,9^. However, identifying and recruiting high numbers of individuals at risk of PD based on distinct NMS, such as isolated RBD (iRBD) and/or hyposmia, has proven inefficient for various reasons and a larger screening approach is needed. PredictPD UK was the first project to use an internet-based strategy to screen a target population for people at risk of PD^10^. Participants were primarily recruited through support groups such as Parkinson’s UK. Cost-effective population-based approaches that screen for PD risk factors in larger populations are currently unavailable, and a comprehensive population-based screening initiative that allows for a better understanding of the prevalence of NMS in the general population is also lacking. Healthy Brain Ageing (HeBA) is a multi-center, European initiative to address these unmet needs.

Among the HeBA centers, Kassel is unique due to Germany’s rigorous municipal registry of inhabitants, which allows for a targeted population outreach. The other European centers each use different outreach strategies. Innsbruck, Austria (Principal Investigator (PI): Werner Poewe and Philipp Mahlknecht), Barcelona, Spain (PI: Maria J Marti and Eduardo Tolosa), and Luxembourg; Luxembourg (PI: Rejko Krüger) rely on media campaigns. As their approaches are less population-based than Kassel’s, they will be reported separately.

The aim of our HeBA Kassel study is to use an online questionnaire on risk factors for PD and/or dementia to identify people with prodromal PD. This prospective, population-based recruitment approach focuses on the elderly in a geographically defined area (City and District of Kassel/Germany) to support future neuroprotective disease strategies.

## Results

**Fig. 1:**
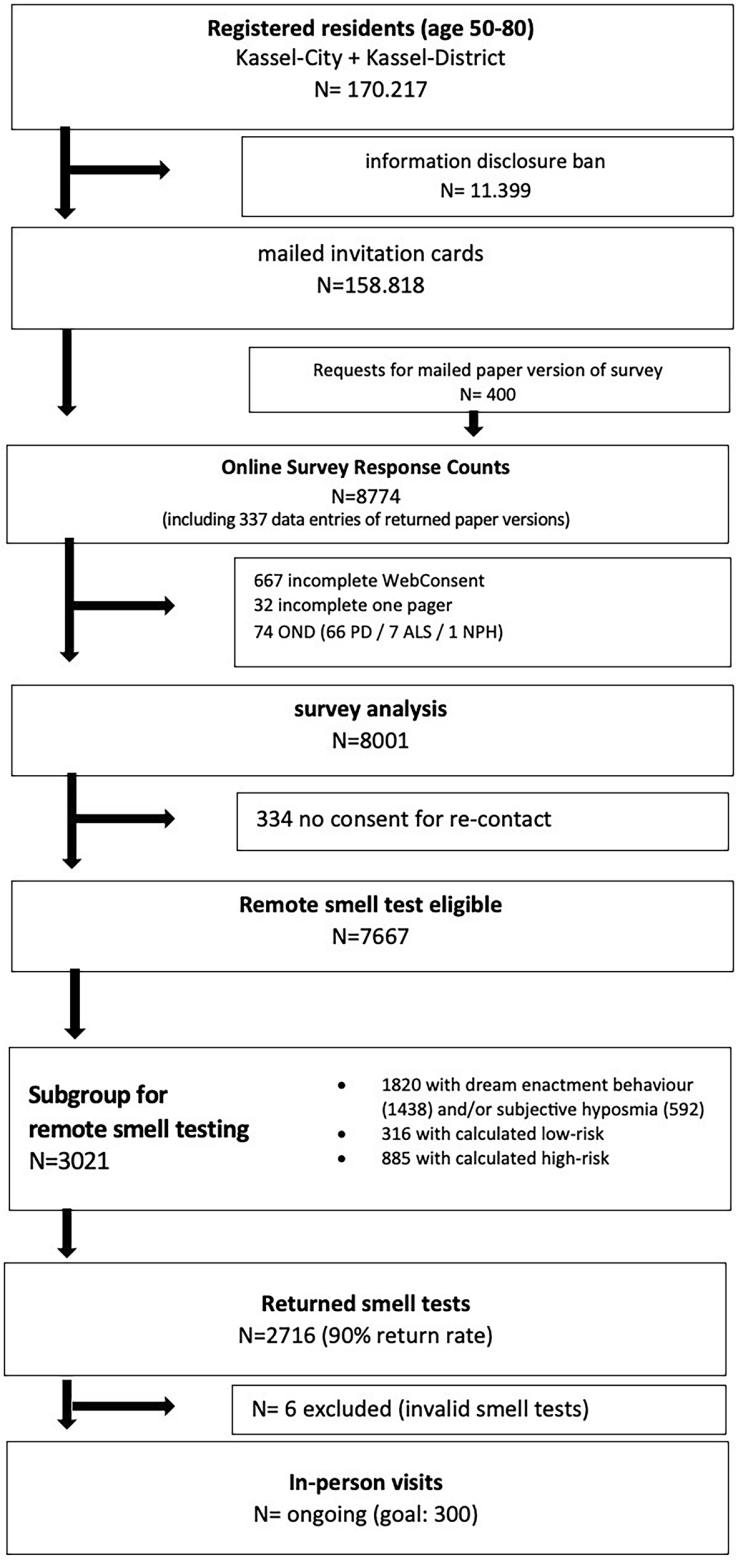
Kassel study flowchart. N number of participants, OND other neurological diseases, PD Parkinson’s disease, ALS amyotrophic lateral sclerosis, NPH normal pressure hydrocephalus, *one pager* includes items for year of birth, sex and five high-interest questions.

The Kassel study flowchart is pictured in Fig. 1. Financial aspects of conducting the study are given in Supplementary Table 1.

### Outreach

The City and District of Kassel cover ~1400 km^2^ (540 miles^2^) and have ~445,000 inhabitants. As of December 2021, 170,217 inhabitants between the ages of 50 and 80 were registered at 29 local registry offices. Of these, 11,399 had an information disclosure ban in effect (details in Supplementary Table 2]). We obtained the postal addresses of 158,818 residents to whom we sent personalized invitation cards. After a test batch of ~10,000 cards on 18 January 2022, six consecutive batches of ~25,000 mailings were sent every three to four weeks between 11^th^ February and 9^th^ June 2022. Each batch consisted of addresses of a subdivision of the geographical area sorted by postcodes. A timeline of online response upon mailed invitations is shown in Supplementary Fig. 1.

Between January and July 2022, our hotline staff answered 923 phone calls and 206 e-mails. Reasons for contacting our staff were “no internet access” (27%), “trouble accessing the homepage” (24%), “survey-related questions” (9%), “no reception of invitation card” (17%), “data protection queries” (5%), “lost invitation card” (4%).

Upon request, we sent 400 paper versions of the online survey to invited participants, with a return rate of 84% (*n* = 337).

### Online Survey

Demographic characteristics of online survey participants can be found in Table 1: Overall, 8001 online surveys were considered for further analysis (after excluding those with invalid web consent, incomplete high-interest questions (HIQ), or with a history of relevant other neurological diseases). This corresponds to 5% of the contacted population, 59.4% were women with an average age of 65 years.

**Table 1:** Demographic characteristics and results of the high-interest questions (HIQ) and all questionnaires of online survey participants recruited by HeBA Kassel

HeBA Kassel, N = 8001	Characteristic	Count or mean	Percentage or SD
***Sex***	Men	3247	41%
	Women	4750	59%
	Diverse	4	0.05%
***Age***	Age (in years)	64.65	7.86
***Educational Duration***	Years of Education	15.25	4.61
***Educational Status***	Education -Primary	703	9%
	Education -Secondary	898	11%
	Education -Further/A-Level	3283	41%
	Education -Higher education	3025	38%
***Living*** ***Situation***	Living alone	1565	20%
	Living with partner	5982	75%
	Living with other family members	1653	20%
	Living with professional aid	27	0.34%
*Calculated* ***probability for prodromal PD*** *according to MDS criteria*^11^	<1%	4223	53%
	1-5%	2976	37%
	5-15%	557	7%
	15-30%	142	2%
	>30%	97	1%
***HIQ Family history of PD*** *Does anyone in your family have Parkinson’s (a blood relative)?*	PD in Family: No	6538	82%
	PD in Family: Yes	985	12%
	PD in Family: Unknown	478	6%
***HIQ Family history of AD*** *Does anyone in your family have Alzheimer’s disease (a blood relative)?*	AD in Family: No	5929	74%
	AD in Family: Yes	1171	15%
	AD in Family: Unknown	901	11%
***HIQ Subjective hyposmia*** *Do you have any problems with your sense of smell? (Please consider ONLY long-term difficulties, NOT temporary problems, such as during a viral infection.)*	No	7348	92%
	Yes	653	8%
***HIQ Dream enactment behavior*** *Do you sometimes have very vivid dreams OR have you ever been told, or suspected yourself, that you seem to “act out your dreams” while asleep (for example, punching, flailing your arms in the air, making running movements, etc.)?*	No	6805	85%
	Yes	1196	15%
***HIQ Subjective memory impairment*** *Do you think your memory is bad or even very bad as compared to your peers?*	No	6852	86%
	Yes	1149	14%
***Schechtman’s Physical Activity Single Question (PASQ)*** *Do you currently participate in any regular activity or program (either on your own or in a formal class) designed to improve or maintain your physical fitness?*	Unanswered	167	2%
	No	2548	32%
	Yes	5286	66%
***Geriatric Depression Scale (GDS)***	Negative (total score <5)	6764	85%
	Positive (total score ≥5)	1237	15%
***Non-Motor Symptoms Questionnaire (NMSQ)***	Mean total score	4.85	3.83
***MDS-UPDRS Part IB*** *(non-motor aspects of daily living)*	Mean total score	4.51	3.67
***MDS-UPDRS Part II*** *(motor aspects of daily living)*	Mean total score	1.52	3.02
***Penn Parkinson’s Daily Activities Questionnaire-15 (PDAQ-15)*** *Total Score*	37 - 43	16	0.2%
	<37 (cutoff dementia)	20	0.3%
	>43 (cutoff non-demented/MCI):	7728	97%
	Unanswered	237	3%

*PD* Parkinson’s disease, *AD* Alzheimer’s disease, *HIQ* high-interest question, *MDS* International Parkinson and Movement Disorder Society, *UPDRS* Unified Parkinson’s Disease Rating Scale, *SD* standard deviation, *MCI* mild cognitive impairment.

Results of the HIQ and all questionnaires are shown in Table 1: 12% indicated a positive family history of PD, 14.6% for Alzheimer’s disease (AD) (11.2% of first degree). Subjective hyposmia was declared by 8%, dream enactment behavior by 15%, and subjective memory impairment by 14%.

### Risk ranking

**Fig. 2:**
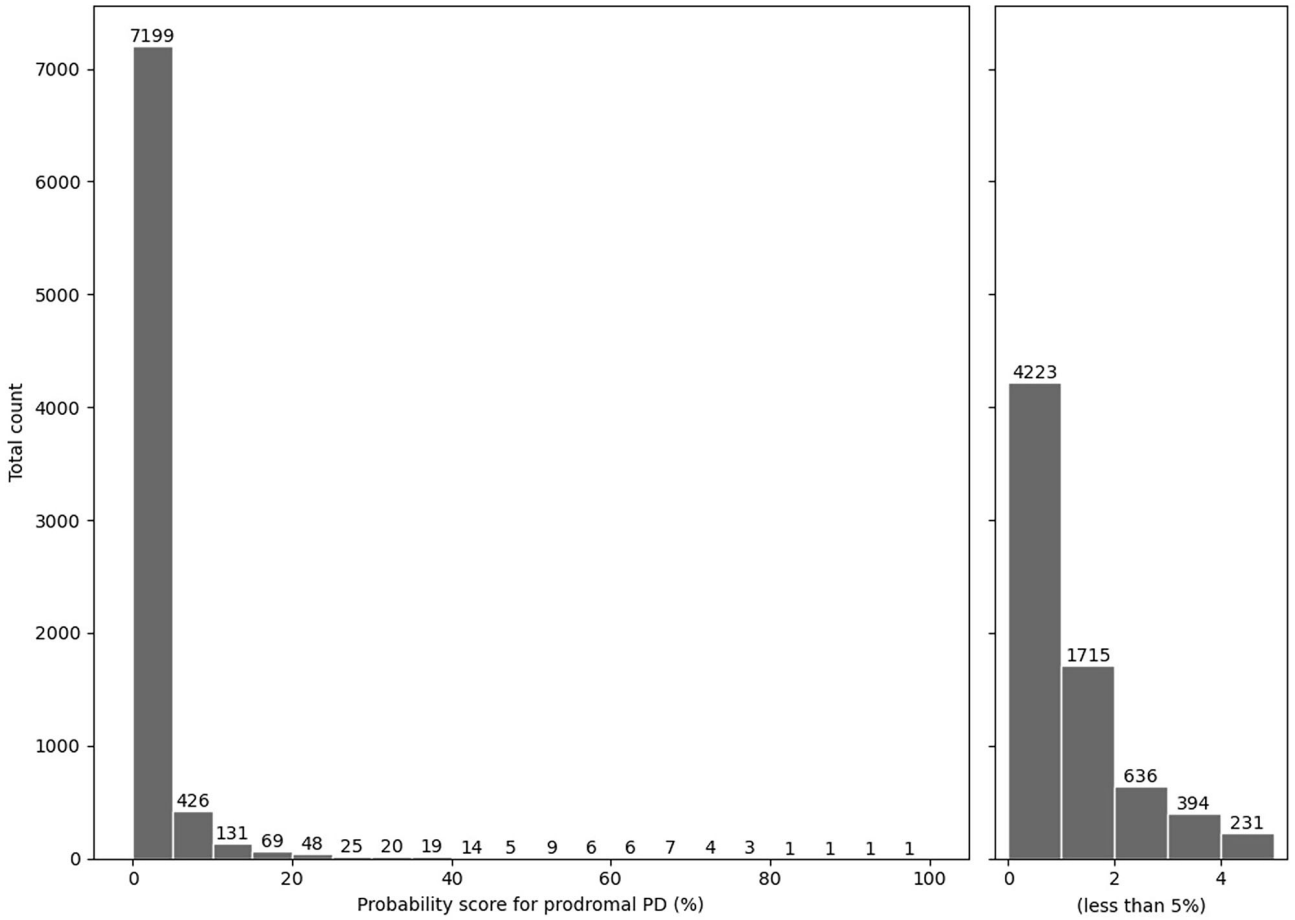
Distribution of the calculated probability for prodromal Parkinson’s disease according to MDS criteria. *x*-axis: calculated probability score in percent according to MDS criteria (Heinzel et al.^11^) in 5% intervals for the entire cohort (left part) and 1% intervals for the cohort below 5% probability score (right part); y-axis: total count of participants with the respective probability score; *MDS* International Parkinson and Movement Disorder Society.

The distribution of the calculated probability score for prodromal PD according to Heinzel et al.^11^ is shown in Fig. 2. Four participants scored a probability of >80%, 39 participants >50%, and 370 > 10%, respectively. >50% of the participants scored below 1% probability.

Results of risk-ranking relevant items are summarized in Supplementary Table 3: Depression/anxiety (based on a self-reported diagnosis and/or a Geriatric Depression Scale (GDS) Score >5) was the most frequently applied positive likelihood ratio for a prodromal symptom (24%). This was followed by dream enactment behavior based on the single dream enactment HIQ or a positive Innsbruck RBD Inventory (RBD-I) result (21%). Urinary dysfunction was considered a risk factor in 11% of the entire cohort (defined as men who answered positively to the respective items of both the Non-motor symptoms Questionnaire (NMSQ) and the International Parkinson and Movement Disorder Society (MDS)-Unified PD Rating Scale (MDS-UPDRS)). Additionally, 9% of the cohort reported a positive first-degree family history of PD.

### Smell assessment

A total of 3021 University of Pennsylvania Smell Identification Tests (UPSIT) were sent between 18^th^ March 2022 and 13^th^ March 2023, of which 2382 were returned within 21 days (immediate return rate 79%). 104 UPSITs could not be delivered or were returned unanswered. We sent 529 reminders, thereafter another 334 UPSITs returned (63% reminder response rate). In total, we received 2716 UPSITs (overall return rate 90%), of which six were excluded as “invalid UPSIT” (see methods section). Characteristics of the UPSIT respondents are given in Table 2. Results of the UPSIT, stratified according to the age- and sex-specific percentile, are given in Table 3: Participants with UPSIT results below the 15^th^ percentile were significantly more likely to be women, younger, and to report subjective hyposmia and subjective memory impairment (*p* < 0.01). Without reaching statistical significance, those below the 15^th^ percentile had higher MDS-UPDRS sub-total scores (mainly part II for motor aspects of experiences of daily living), as well as higher NMSQ total score and GDS (≥5), and had lower Penn Parkinson’s Daily Activities Questionnaire (PDAQ) total scores. Applying a more rigorous recently suggested^12^ definition of smell dysfunction—a UPSIT result ≤9th age- and sex-specific percentile—consolidates our findings and the MDS-UPDRS part II sub-total score also reaches statistical significance (*p* < 0.01) [Supplementary Table 4].

**Table 2:** Characteristics including results of the high-interest questions (HIQ) of smell test respondents

*Characteristic*	HeBA Kassel, *N* = 2710		
*Sex*	Women	1626	60%
	Men	1084	40%
	Other	0	0%
*Age*	64.83 (8.31)		
*Years of education*	15.30 (5.50)		
*Educational degree*	Primary	244	9%
	Secondar*y*	312	12%
	Higher education	1016	38%
	Further/A-level	1127	42%
*Living* *situation*	Living alone	550	20%
	Living with partner	2041	75%
	Living with other family members	572	21%
	Living with professional aid (e.g. nursing home)	11	0.4%
*Calculated probability for prodromal PD according to MDS criteria*^11^ *derived from Online Survey responses*	<1%	941	35%
	1–5%	1150	42%
	5–15%	427	16%
	15–30%	117	4%
	>30%	75	3%
*HIQ Dream enactment behavior*	No	1733	64%
	Yes	977	36%
*HIQ Subjective hyposmia*	No	2197	81%
	Yes	513	19%
*HIQ Family history of PD*	No	2330	86%
	Yes	380	14%

Presented values are Count and Count Percentage or Mean (Standard Deviation).

*PD* Parkinson’s Disease, *MDS* International Parkinson and Movement Disorders Society, *HIQ* high-interest question (for the wording of the HIQ please refer to Table 1).

**Table 3:** Characteristics of respondents below and above the 15th (age- and sex-adjusted) percentile of the smell test

Clinical characteristics	≤15th%ile *Count or Mean*	≤15th%ile *Count % or SD*	>15th%ile *Count or Mean*	>15th%ile*Count % or SD*	*p*-value *(2tailed)*
***N***	1019	38%	1691	62%	
***Sex (men)***	292	29%	792	47%	**<0.01**
***Age***	62.74	8.20	66.09	8.12	**<0.01**
***HIQ Family history of PD***	132	13%	248	15%	0.50
***HIQ Subjective hyposmia***	296	29%	217	13%	**<0.01**
***HIQ Subjective memory impairment***	222	22%	291	17%	**<0.01**
***HIQ Dream enactment behavior***	379	37%	598	35%	0.33
***MDS-UPDRS Part IB*** *total score*	5.69	4.21	5.48	4.07	0.20
***MDS-UPDRS Part II*** *total score*	2.32	4.04	2.04	3.27	0.05
***NMSQ*** *total score*	6.42	4.59	6.13	4.19	0.12
***PDAQ-15*** *total score*	68.99	6.69	69.28	7.75	0.32
***GDS*** *score >=5*	235	23%	332	20%	0.23

*HIQ* high-interest question (for the wording of the HIQ please refer to Table 1), *PD* Parkinson’s disease, *MDS* International Parkinson and Movement Disorders Society, *UPDRS* Unified Parkinson’s Disease Rating Scale, *NMSQ* Non-Motor Symptoms Questionnaire, *PDAQ-15* Penn Parkinson’s Daily Activities Questionnaire-15, *GDS* Geriatric Depression Scale, *SD* standard deviation, age- and sex-adjusted percentile according to Brumm et al.^28^.

## Discussion

Population-based data on risk factors for PD are important for estimating the number of potential participants for upcoming prevention trials. The HeBA Kassel study is important for future enrollment considerations because of its unique and personalized outreach to every registered elderly inhabitant in a geographically defined area. The data from HeBA Kassel broaden our understanding of the prevalences of signs and symptoms of prodromal PD stages in the real world. We were able to show that a stepwise approach with an online questionnaire followed by remote smell testing is a feasible strategy for identifying individuals in the general population who may be at risk for prodromal PD.

We are confident that the HeBA Kassel cohort is a population that is as close as possible to a real-world setting despite the following limitations that may have caused selection bias: In general, the elderly population is less prone to take part in online surveys and some individuals lack the technological requirements for completing such a survey. However, in recent years the number of people without the necessary technology has declined: according to the 2022 microcensus of the Federal Statistical Office of Germany, 77.1% of German inhabitants between the ages 65 and 75 have used the internet within the past 3 months (compared to 56% in 2018)^13^. We sought to overcome technological limitations by allowing prospective participants to request a paper version of the survey (400 requests were made). An information disclosure ban was in effect for 11,399 people, which may have led to the systematic exclusion of specific demographic groups, although most of these individuals would likely not have been eligible for any real-world population screening.

Cognitive decline in the elderly might have been a further limiting factor for participating in the study. Even though approximately 14% of the entire cohort declared subjective memory impairment compared to their peers, the entire drop-out rate throughout the online survey was low (approx. 8% with the majority at the initial web consent page). Functionally relevant cognitive deficits might have been a beneficial barrier for our study’s purpose by excluding those who were cognitively incapable of participating in an online survey and/or remote smell testing, and who, therefore, may have already met the criteria for dementia. Furthermore, our sample seems to over-represent those with higher educational attainment. While higher education may be advantageous for retention in prospective observational studies and may increase motivation to participate in preventive trials, it could also be a disadvantage as these participants may be more likely to lead healthier lifestyles that are known to be preventive for PD. Nevertheless, this potential educational selection bias should be taken into account when designing similar studies, especially in regions around the world that have lower average education levels.

Depressive symptoms, anxiety, and apathy might have been limiting factors preventing people from taking part in the study. This is relevant since these symptoms could be NMS in prodromal PD^14^. Approximately 15% of respondents were positive on the depression screening instrument (Geriatric depression scale), which is higher than the results of the German National Cohort (NAKO), a population-based mega cohort^15^ where the authors found between 5.5% and 9.3% of participants positive on the Depression Scale of the Patient Health Questionnaire (PHQ-9) among the ages 50–72. One could argue that depressive symptoms in our study might have been influenced by the COVID-19 pandemic, which occurred between 2020 and 2023 in Germany. Our study started in January 2022. Nevertheless, the first emerging studies show largely unaltered mental well-being in the German elderly population during that period^16^. Another source of selection bias could be a positive family history of neurodegenerative diseases and a fear of being genetically at risk. To reduce this risk of selection bias, the study name was chosen not to include illness-related wording, but to focus on salutogenesis: Healthy Brain Ageing (German: Gesund Altern). In all participant-facing materials, words like Alzheimer’s and Parkinson’s were avoided and we used the expression “age-related disorders of the brain”. In contrast to other studies in the field that used outreach strategies to recruit participants through special interest groups, like PredictPD^10^ or the Parkinson's Progression Markers Initiative Online Study (PPMI Online)^17^, the number of participants with a positive family history for PD or AD in HeBA was lower and in a range (8.8% positive first-degree family history of PD, 11.2% for AD, respectively) expected from the prevalence of these diseases in the target population. Even though participants with a positive first-degree family history for PD were enriched in our subset of UPSIT respondents (14.12%) compared to the entire online respondent’s cohort, this risk factor was not significantly different between hyposmic and normosmic individuals according to their remote UPSIT results.

The major strength of our study is that we identified >1000 individuals with hyposmia (defined as below the 15th age- and sex-stratified percentile UPSIT) with only 3000 remote smell tests (of which 300 were sent to controls of “low risk”). Even if we were more rigorous by applying the 9th age- and sex-specific percentile as a threshold for anosmia, we still identified 530 individuals. There is profound evidence that smell loss is an important early sign of neuronal synuclein diseases (such as prodromal PD), which is further supported by an association between incidental Lewy bodies in autopsy studies and olfactory dysfunction^18^. Presence at autopsy of Lewy bodies increases with age and can be present in up to 20% of brains and 26% of olfactory bulbs in the elderly^19,20^. Further in-patient investigations, DaT-SPECT imaging, and longitudinal follow-up will help us understand the natural course of these hyposmic individuals. Even though our recruitment strategy was successful, substantial manpower and funds are needed to reproduce this effort. Possible strategies to reduce costs are provided in Supplementary Table 1 for the interested reader^21^.

Our initial plan was to select individuals for remote smell testing according to their calculated probability score for prodromal PD according to MDS criteria. However, we identified only a few individuals with a very high probability score, even though some self-reported subjective signs (like hyposmia) are indicated earlier in prodromal PD than detected by clinical assessments^22^. Additionally, a range of prediagnostic features is detectable several years before diagnosis of PD in primary care^23^. Conversely, the lead time for subtle motor symptoms might be quite short and difficult to detect in people with prodromal PD^24^. Most high-likelihood-ratio items from the MDS algorithm cannot be assessed remotely, while those that can be captured online have rather low likelihood ratios. Since the start of our study independent groups have reported similar findings. For example, a prospective validation study demonstrated suboptimal sensitivity in their population-based random sample^25^. Another group was unable to predict conversion to PD in the general population using a similar set of MDS criteria^26^. Furthermore, some of the original authors of the MDS algorithm concluded that most PD patients will not meet these criteria before diagnosis^27^ and the updated version of the criteria did not significantly improve sensitivity^24^.

When interpreting predictors for a low smell test result in our data, it should be emphasized that individuals were ultimately selected for remote smell testing primarily based upon reporting subjective hyposmia/or dream enactment behavior, which introduces some selection bias. This could explain some of the significant differences in subjective hyposmia between hyposmic and normosmic participants according to their UPSIT results. On the other hand, self-reported olfactory loss occurs approximately 2 years earlier in prediagnostic PD than detected by clinical assessment^22^.

Notably, despite the selection bias leading to an enrichment of participants with dream enactment behavior, this risk factor was not different between hyposmic and normosmic individuals. Interestingly, total scores of the MDS-UPDRS part II for motor aspects of experiences of daily living were significantly worse in anosmics (≤9th percentile), possibly demasking some subtle motor and cognitive signs in this group. Surprisingly, scales assessing NMS (NMSQ, MDS-UPDRS part Ib, PDAQ, GDS) showed only non-significant trends towards worse results in people with hyposmia. Unexpectedly, individuals with hyposmia in our cohort were significantly younger than those with normal olfaction. This may reflect a motivational participation bias, enriching the younger group with individuals who have self-perceived health concerns. However, a more likely explanation is the recently updated age- and sex-specific UPSIT norms^28^, which seem less precise in younger populations, especially women, thereby potentially overestimating smell deficits. Notably, the normative data for percentile values were based on approximately 550 women and 200 men in each age category under the age of 60. Hopefully, the future integration of more participants from other HeBA centers will increase the power to further investigate all above mentioned findings and will also help delineate individual items of the compound scores.

HeBA Kassel is a new population-based cohort that contributes to increasing efforts to identify people with prodromal PD before they develop manifest motor disease. Some aspects of our study can be compared to international efforts while others are unique to our cohort. For instance, PredictPD UK, the Parkinson Associated Risk Syndrome study (PARS), or PPMI Online^17^ use online or remote mailing assessments in the general population with an outreach mainly through patient or special interest groups as well as media campaigns. This differs from the direct recruitment approach taken by HeBA Kassel. PredictPD recruited 1323 online participants^10^; PPMI Online 5776 participants who were eligible for remote smell testing (>age 60, without a diagnosis of PD, as of January 2024, recruitment ongoing)^12^; and PARS included 4999 participants who completed remote smell testing^14^. These efforts are comparable to the recruitment strategies taken by other European HeBA centers. Other more community-based cohorts, like the Bruneck study (*n* = 934)^29^, or the Honolulu-Asia Aging Study (HAAS) (*n* = 8006)^4^, contribute to our understanding of prevalences of certain symptoms/risk factors. However, these began in the last century, long before it was possible to use online recruiting methods and before it was possible to envisage preventive trials like Path-to-Prevention (P2P^9^).

In Germany, the Cologne RBD study^30^ recruited 78 participants with iRBD through newspaper advertisements and a stepwise screening process that concluded with a polysomnography. While the Tübinger Evaluation of Risk Factors for Early Detection of Neurodegeneration (TREND) recruited participants using a similar media campaign, 493 of 715 participants had at least one selected prodromal marker (lifetime depression, RBD, impaired olfaction)^31^.

In summary, a direct recruitment approach for online surveys is a feasible strategy for selecting elderly individuals to participate in remote smell testing and for identifying those with hyposmia or even anosmia who might be at risk for prodromal PD. Thirty-eight percent of our UPSIT respondents were objectively hyposmic, which exceeds the expected prevalence of prodromal PD in the general population. This highlights the effectiveness of our selection strategy in enriching the sample for individuals with hyposmia. Some features like subjective hyposmia, subjective memory impairment, or subtle motor scores might predict pathological smell test results. Further analysis—by integrating results from other HeBA centers and our ongoing in-person visits—will improve the recruitment strategy and optimize the online survey for predicting (a) pathological smell test results, (b) pathological DaT-SPECT, (c) prodromal PD individuals and (d) ultimately progression to manifest PD. These future analyses should integrate the ongoing in-person visits, biosample results, and comparisons to the other HeBA initiative centers that use different recruitment strategies.

## Methods

HeBA Kassel consists of the following phases: individual outreach and online survey (phase 1a); the recontact of selected individuals for a remote smell assessment (phase 1b); and in-patient visits of high- and low-risk individuals in the clinic (phase 2); and planned follow-up of high-risk individuals (phase 3). Phases 2 and 3 are on-going. Here we present the results of phases 1a and 1b.

### Online survey (phase 1a)

To evaluate risk factors and prodromal symptoms of PD, we created a survey that included demographic questions [year of birth (YOB), sex, height, weight, handedness, occupational/educational levels, and living situation], questions about exposure to drugs, alcohol, nicotine, caffeine, tea, solvents or pesticides, as well as medical history (medical conditions based on Charlson Comorbidity Index^32^, current medication as a free text entry field, COVID-19 history) and family history (for PD and AD). Most of these questions were adapted from the Predict PD UK study^10^ and translated by the Mapi Institute (New Linguistic Validation Manual For Health Assessment, mapi-institute.com) using a professional linguistic validation process (including two forward translations and one backward translation). Additionally, validated German translations of several questionnaires were incorporated: the Geriatric Depression Scale (GDS^33^), the Non-motor symptoms Questionnaire (NMSQ^34^), the International Parkinson and Movement Disorder Society-Unified PD Rating Scale (MDS UPDRS^35^) parts Ib and II, and the Innsbruck RBD Inventory (RBD-I^36^). The Penn Parkinson’s Daily Activities Questionnaire-15 (PDAQ15^37^) and Schechtman’s Physical Activity Single Question (PASQ^38^) were translated into German and validated by the Mapi Institute as described above. To capture a minimum essential dataset per individual—even if the entire questionnaire was not completed—the first page of the online survey included the YOB and sex as well as five single high-interest questions (HIQ) on family history of PD and AD, subjective hyposmia, dream enactment behavior (modified from the RBD Single-Question Screen^39^) and subjective memory impairment.

### Outreach

For phase 1a, the local population of the city and the district of Kassel/Germany, comprising 170,000 inhabitants aged between 50 and 80, was invited to take part in the Online Survey. Using a population-based recruitment approach, all registered residents of the Kassel area were contacted individually by post. They received a personalized card with an invitation, which included a weblink and a unique participation code (token) for the online survey [Supplementary Fig. 2]. Postal addresses were obtained from the local Residents’ Registration Office (in Germany, all inhabitants need to register at this municipal office). Some information (like name, age, and address) can be accessed for non-profit/charitable use. This was the case for the Healthy Brain Ageing study unless an inhabitant opted out by requesting a data access restriction (information disclosure ban details in Supplementary Table 2). Along with the individual outreach, the study was promoted through a newspaper advertising campaign, articles in local magazines, posters and flyers in physicians’ offices, and public talks on healthy brain aging.

Invited participants who reached out to the HeBA study team because they were unable to complete the online survey were provided with a paper version of the questionnaire and a prepaid return envelope. The data on these forms were extracted by the HeBA study team. Individuals who lost their personal invitation card could contact the HeBA local telephone hotline to request a new card and token to be sent by post.

### Risk assessment

Data from the completed questionnaires enabled a risk score to be calculated by a baseline pretest (i.e., “prior”) probability of disease (estimated age-adjusted prevalence of prodromal PD) and then adding up the relative risk or odds ratios [Supplementary Table 5] associated with each response to arrive at a post-test probability of disease. These scores were derived from studies on prodromal PD and its risk factors^11^ (see [Supplementary Table 3] for details on how the algorithm was applied; in brief, a conservative approach for applying likelihood ratios was used). Everyone who completed the items YOB, sex, and all five HIQ were ranked according to their individual probability of prodromal PD.

We excluded from the risk calculation participants who did not provide web consent or who had an established diagnosis of PD or other severe neurological disease (e.g. amyotrophic lateral sclerosis, progressive supranuclear paresis, multiple system atrophy, AD, Lewy body dementia, corticobasal syndrome, frontotemporal dementia, Huntington’s disease, Wilson’s disease).

### Smell assessment (Phase 1b)

Phase 1b consisted of remote smell assessments for a subset of online survey participants (phase 1a). According to the online survey risk ranking, individuals categorized as high, medium, and low risk received a University of Pennsylvania Smell Identification Test (UPSIT^28^) via post together with detailed instructions for completing the test and a prepaid return envelope. In addition, an UPSIT was sent to everyone who reported subjective hyposmia or dream enactment behavior in the HIQ. Participants were instructed to return the tests within 21 days; if the UPSIT was not returned within this timeframe a reminder was sent.

Anyone undergoing treatment with Parkinsonism-inducing drugs was excluded from the UPSIT mailing list. These drugs included typical neuroleptics (phenothiazine/chlorpromazine, butyrophenone/haldol, diphenylbutylpiperidine/pimozide, benzamine/sulpiride), atypical neuroleptics (risperidone, olanzapine, aripiprazole), as well as reserpine, tetrabenazine, metoclopramide, verapamil and flunarizine. The free text entry fields for current medication of the online survey were screened manually for those exclusionary drugs.

Returned UPSITs were assessed centrally by the HeBA study team and data were entered into a RedCap Database. Since only one answer per item is correct, any items with no or more than one answer were considered invalid. We adopted a very conservative approach: invalid answers were counted as correct answers unless more than three items were invalid. In such cases, the entire assessment was defined as invalid (“invalid UPSIT”). Hyposmia was defined by a UPSIT score ≤15^th^ age- and sex-specific percentile^28^.

### In-Person visits (phase 2)

Phase 2 is currently on-going. Subsets of high, medium, and low-risk participants are being invited for in-person visits. During these visits, their risk is assessed using in-depth investigations for single risk factors or prodromal symptoms (neurological exam, substantia nigra ultrasound, supine and upright blood pressure measurements etc.) and future follow-up visits will determine conversion to manifest PD. Subgroups are sent to DaT-SPECT imaging and receive SAA testing.

### Standard protocol approvals, registrations, and patient consent

HeBA Kassel was approved by the local ethics committee (Ethik-Kommission bei der Ländesärztekammer Hessen, 2019-1395-evBO). The study was performed in accordance with the Declaration of Helsinki. HeBA is registered in the German clinical trials register (DRKS00025979). Electronic consent was obtained from all phase 1 participants, additional written informed consent was obtained from all phase 2 participants.

## Supplementary information

Supplement Material blackened

## Data Availability

All data generated or analyzed during this study are included in this published article and its supplementary information files. Additional information can be provided upon reasonable request to the HeBA data access committee.
