# Supplementary material for: Identifying individuals at-risk of developing Parkinson’s disease using a population-based recruitment strategy: The Healthy Brain Ageing Kassel Study
Source: NPJ Parkinsons Dis. 2025 Jul 18;11:216. doi: 10.1038/s41531-025-01008-w (PMC12274381; doi:10.1038/s41531-025-01008-w)
Supplement: Supplementary file 1 — Supplement Material blackened [file 41531_2025_1008_MOESM1_ESM.pdf]

Supplement Material

Main Document: Identifying individuals at-risk of developing Parkinson's disease using a population-based recruitment strategy: The Healthy Brain Ageing Kassel Study

Supplement Table 1: Financial aspects of the study

**Supplement Table 1: Financial aspects of the study** *The following costs occurred while conducting the study and would have to be taken into account when reproducing this recruitment strategy, applying local fees, taxes and salaries (detailed amounts for Germany may be requested from the corresponding author).*	
Regulatory costs	• Ethics Committee / Institutional Review Board fees<br>• Data Protection Authority fees<br>• Residents' Registration Office fees
Invitation card logistics	• Printing, labeling, postage, mailing
Accompanying media campaign	• Flyers, posters, local newspaper advertisement
Remote smell test logistics	• Smell test purchase<br>• Labeling, postage (including prepaid return envelopes), mailing
Information technology (IT)	• Infrastructure establishment and maintenance<br>• Website / domain with online survey platform<br>• Database software
Personnel costs	• Principal investigator (5%)<br>• Study physician (50%)<br>• Study coordinator, study nurse (50%)<br>• UPSIT coordinator, study nurse (50%)<br>• IT administrators (cumulative positions of 75%)<br>• Several part-time jobs for the hotline and mailing logistics (cumulative positions of 100%)

Strategies to reduce costs:

In the Healthy Brain Ageing (HeBA) Kassel cohort, integrating various detailed instruments into the online questionnaire to cover all remotely assessable items of the MDS probability algorithm was unhelpful. Considerably reducing the size of the online questionnaire would, in turn, lower IT-related costs. Once recruitment at the other European HeBA centres is finalized—thereby increasing our statistical power (four-fold sample size)—we will propose a substantially shortened version of the questionnaire. A recent example of such a simplified recruitment strategy is called Smell Test Direct (ST Direct, *Brown et al. 2025*), where community members are directly invited to complete a smell test. Furthermore, shorter versions of remote smell test instruments currently in development might be less expensive (*Li et al. 2024*). Using an online platform for remote data entry of smell test results could also reduce costs related to the return envelopes and manual data input. Finally, we recommend sharing IT costs with other centers, as successfully done within the European HeBA consortium.

Brown, E. G. et al. Staged Screening Identifies People with Biomarkers Related to Neuronal Alpha-Synuclein Disease. Ann. Neurol. 97, 730-740 (2025).

Li, J. et al. Development of a Simplified Smell Test to Identify Patients with Typical Parkinson's as Informed by Multiple Cohorts, Machine Learning and External Validation. medRxiv, 2024.2008.2009.24311696 (2024).

Supplement Table 2: Information disclosure ban

**Supplement Table 2: Information disclosure ban**<br>According to German federal laws and regulations, information disclosure bans are applicable:	
Upon reasonable request<br>(§ 51 BMG)	If a person could be at risk of harm to life, health, personal freedom or similar interests worthy of protection as a result of information from the register
Mandatorily<br>(§ 63 PStG, § 1758 BGB)	For transsexuals as well as for cases of adoption or adoption foster care relationships
Upon request<br>(§ 52 BMG)	For persons who are registered as residents in certain facilities (nursing homes, facilities for protection against domestic violence, facilities for the treatment of addictions)
BMG: Bundesmeldegesetz (German Federal Registration Act)<br>*PStG: Personenstandsgesetz (German Civil Status Act)*<br>*BGB: Bürgerliches Gesetzbuch (German Civil Code)*	

Supplement Table 3 - Applied likelihood ratios for calculating the risk probability according to MDS criteria

Risk factor / Prodromal Symptom	HeBA Kassel, N = 8,001
Regular pesticide exposure<br>*"Do you think you have received significant exposure to pesticides in your lifetime?"*	LR+ 196 (2.5%)<br>LR- 7805 (97.6%)
Occupational solvent exposure<br>*"Do you think you have received significant exposure to solvents in your lifetime?"*	LR+ 389 (4.9%)<br>LR- 7612 (95.1%)
Non-use of caffeine<br>*"Do you drink coffee?" "Do you drink tea?"*<br>*(LR-) IF "Number of cups per day?" >1*	LR+(No use) 115 (1.4%)<br>LR1 1541 (19.3%)<br>LR- (Regular use) 6345(79.3%)
Current/Never/Former smoking<br>*(LR+) IF "Do you smoke?" = "No, I have never smoked"*<br>*(LR-) IF "Do you smoke?" = "Yes, I am a current smoker"*<br>*(LR-) IF "Do you smoke?" = "I used to smoke" AND packyears > 1*	LR+ (never) 3433 (42.9%)<br>LR1 219 (2.7%)<br>LR- (current) 782 (9.8%)<br>LR- (former) 3570 (44.6%)
First-degree relative with Parkinson's Disease<br>*"Does anyone in your family have Parkinson's (a blood relative)?"*<br>*"Which family member(s) has/had Parkinson's disease?"*<br>*(LR+) IF: Mother OR Father OR Sister OR Brother OR Twin Sister OR Twin Brother*	LR+ 702 (8.8%)<br>LR- 7299 (91.2%)
Type 2 Diabetes mellitus<br>*"Do you have or ever had any other medical conditions? (Even if it is controlled with medication, diet, or lifestyle changes)"*<br>*(LR+) IF "Diabetes without complications" OR "Diabetes with complications such as eye damage, kidney problems or nerve damage" AND IF "Is the diabetes you mentioned type 2 diabetes?" = "Yes"*	LR+ 493 (6.2%)<br>LR1 82 (1.0%)<br>LR- 7426 (92.8%)
Physical inactivity<br>*(LR+) IF "Over the PAST WEEK, have you usually had trouble doing your hobbies or other things that you like to do?" = "I am unable to do most or all of these activities." (MDS-UPDRS)*	LR+ 50 (0.6%)<br>LR1 2678 (33.5%)<br>LR- 5273 (65.9%)

(LR-) IF "Do you currently participate in any regular activity or program (either on your own or in a formal class) designed to improve or maintain your physical fitness?" = "YES"	
Dream enactment behavior<br>*(LR+) IF "Do you sometimes have very vivid dreams OR have you ever been told, or suspected yourself, that you seem to "act out your dreams" while asleep (for example, punching, flailing your arms in the air, making running movements, etc.)?" = "Yes" OR IF positive Innsbruck-RBD-Inventory*	LR+ 1694 (21.2%)<br>LR1 2 (0.02%)<br>LR- 6305 (78.8%)
Constipation<br>*(LR+) IF "Have you experienced any of the following in the LAST MONTH? Constipation (less than three bowel movements a week) or having to strain to pass a stool." = YES (NMSQ) AND IF "Over the PAST WEEK have you had constipation troubles that cause you difficulty moving your bowels?" ≧2 (MDS-UPDRS)*<br>*(LR-) "NO" (NMSQ) AND 0 (MDS-UPDRS), respectively*	LR+ 214 (2.7%)<br>LR1 1323 (16.5%)<br>LR- 6464 (80.8%)
Excessive daytime somnolence<br>*(LR+) IF "Have you experienced any of the following in the LAST MONTH? Finding it difficult to stay awake during activities such as working, driving, or eating." = YES (NMSQ) AND IF "Over the PAST WEEK have you had trouble staying awake during the daytime?" ≧2 (MDS-UPDRS)*<br>*(LR-) "NO" (NMSQ) AND 0 (MDS-UPDRS), respectively*	LR+ 262 (3.3%)<br>LR1 4196 (52.4%)<br>LR- 3543 (44.3%)
Symptomatic orthostatic hypotension<br>*(LR+) IF "Have you experienced any of the following in the LAST MONTH? Feeling light-headed, dizzy, or weak standing from sitting or lying." = YES (NMSQ) AND "Over the PAST WEEK have you felt faint, dizzy, or foggy when you stand up after sitting or lying down?" ≧2 (MDS-UPDRS)*<br>*(LR-) "NO" (NMSQ) AND 0 (MDS-UPDRS), respectively*	LR+ 505 (6.3%)<br>LR1 2087 (26.1%)<br>LR- 5409 (67.6%)
Erectile dysfunction (only men)	LR+ 13 (0.16%)

"Please list all medication that you are taking": use of sexuo-pharmaceuticals	
Urinary dysfunction<br>*(LR+) IF "Have you experienced any of the following in the LAST MONTH? A sense of urgency to pass urine makes you rush to the toilet." = YES (NMSQ) AND "Over the PAST WEEK have you had trouble with urine control? For example, an urgent need to urinate, a need to urinate too often, or urine accidents?" ≧2 (MDS-UPDRS) (LR-) "NO" (NMSQ) AND 0 (MDS-UPDRS), respectively*	LR+ (only in men) 847 (10.6%)<br>LR1 3178 (39.8%)<br>LR- (all sex) 3976 (49.7%)
Depression/Anxiety<br>*(LR+) IF GDS Total >5 OR IF "Do you have or ever had any other medical conditions?" = "Depression / Anxiety Disorder"*	LR+ 1888 (23.6%)<br>LR- 6113 (76.4%)
Global cognitive deficit<br>*PDAQ-15 Total Score*	LR+ (PDAQ <37)20 (0.25%)<br>LR1 253 (3.2%)<br>LR- (PDAQ >43) 7728 (96.6%)

Legend: Applied positive (LR+, >1) and negative (LR-, <1) likelihood ratios given as *Count (Count Percentage)* and details on criteria for application are presented along each individual risk factor / prodromal symptom; a neutral likelihood ratio (LR1) was applied if criteria for neither LR+ nor LR- were present; MDS - Movement Disorders Society; UPDRS - United Parkinson's Disease Rating Scale; NMSQ - Non-Motor Symptoms Questionnaire; PDAQ-15 - Penn Parkinson's Daily Activities Questionnaire-15; GDS - Geriatric Depression Scale

Supplement Table 4: Characteristics of respondents below and above the 9th/15th (age- and sex-adjusted) percentile of the smell test

Clinical characteristics	***≤9th%ile*** *Count or Mean*	***≤9th%ile*** *Count % or SD*	***>9th and ≤15th %ile*** *Count or Mean*	***>9th and ≤15th %ile*** *Count % or SD*	***>15th %ile*** *Count or Mean*	***>15th %ile*** *Count % or SD*	*p-value**
N	530		489		1691		
Sex (men)	165	31%	127	26%	792	47%	**<0.01**
Age	62.58	8.44	62.91	7.93	66.09	8.12	**<0.01**
HIQ Family history of PD	69	13%	63	13%	248	15%	0.76
HIQ Subjective hyposmia	200	38%	96	20%	217	13%	**<0.01**
HIQ Subjective memory impairment	118	22%	104	21%	291	17%	0.01
HIQ Dream enactment behavior	199	38%	180	37%	598	35%	0.61
MDS-UPDRS Part IB *total score*	5.89	4.31	5.47	4.10	5.48	4.07	0.125
MDS-UPDRS Part II *total score*	2.58	4.47	2.03	3.47	2.04	3.27	**<0.01**
NMSQ *total score*	6.60	4.82	6.23	4.35	6.13	4.19	0.133
PDAQ-15 *total score*	68.59	7.11	69.41	6.17	69.28	7.75	0.125
GDS *score >=5*	134	25%	101	21%	332	20%	0.47

Legend: HIQ - high-interest question (for the wording of the HIQ please refer to Table 1); PD - Parkinson's Disease; MDS - Movement Disorders Society; UPDRS - United Parkinson's Disease Rating Scale; NMSQ - Non-Motor Symptoms Questionnaire; PDAQ-15 - Penn Parkinson's Daily Activities Questionnaire-15; GDS - Geriatric Depression Scale; SD - Standard Deviation; age- and sex-adjusted percentile according to Brumm et al. 2023; *one-way ANOVA

Supplement Table 5 - Applied likelihood ratios for each risk factor / prodromal symptom according to MDS criteria

**Age**	**pretest probability of disease (estimated age-adjusted prevalence of prodromal PD)**
50-54	0.4%
55-59	0.75%
60-64	1.25%
65-69	2.0%
70-74	2.5%
75-79	3.5%
**Risk factors / Prodromal Symptom**	**Positive (LR+, >1) and negative (LR-, <1) Likelihood Ratio**
Sex	1.2 (men); 0.8 (women)
Regular exposure to pesticide	1.5
Occupational solvent exposure	1.5
Non-use of Caffeine	1.35; 0.88
Smoking	
Current smoker	0.51
Never smoker	1.2
Former smoker	0.91
Family history of Parkinson's Disease	2.5
Diabetes mellitus (type II)	1.5; 0.97
Physical inactivity	1.3; 0.91
Possible RBD (REM sleep behavior disorder)	2.8; 0.89
Constipation	2.5; 0.82
Excessive daytime somnolence	2.7; 0.86
Symptomatic orthostatic hypotension	3.2; 0.80
Erectile dysfunction (men)	3.4; 0.87
Urinary dysfunction	2.0; 0.90
Depression (+/- anxiety)	1.6; 0.88
Global cognitive deficit	1.8; 0.88

Supplement Figure 1: Timeline of online responses to each mailing batch of invitation cards

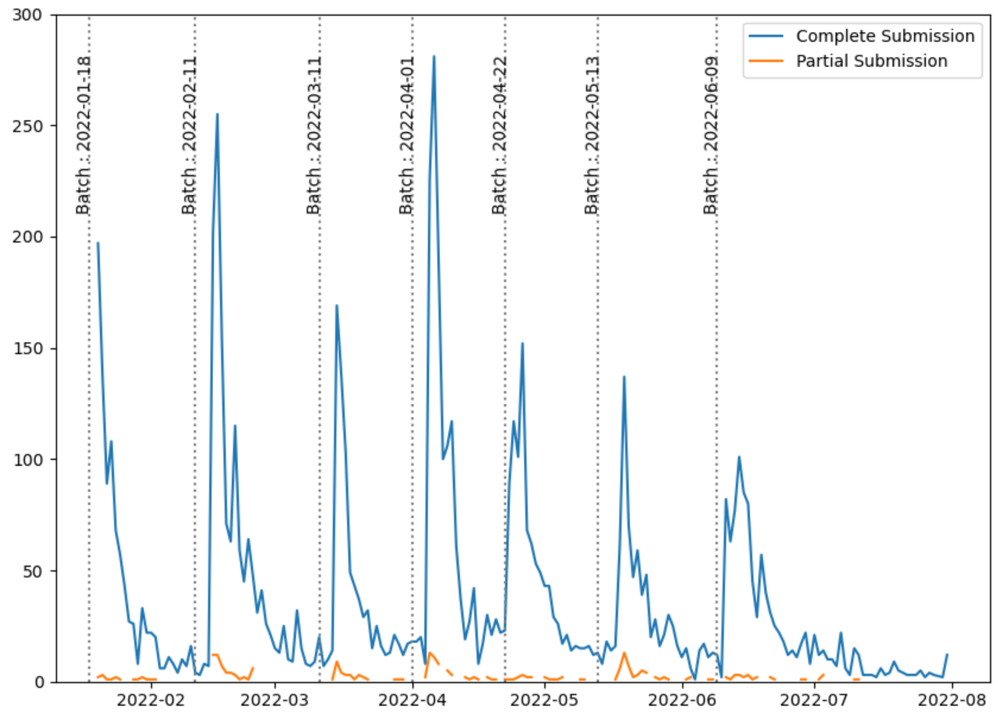

Supplement Figure 1 **Timeline of online responses to each mailing batch of invitation cards.** Timeline of complete (blue line) and partial (orange line) online responses to each mailing batch (vertical black dashed line) of invitation cards. x-axis: time, y-axis: counts of online submissions

Supplement Figure 2: Sample of the invitation card

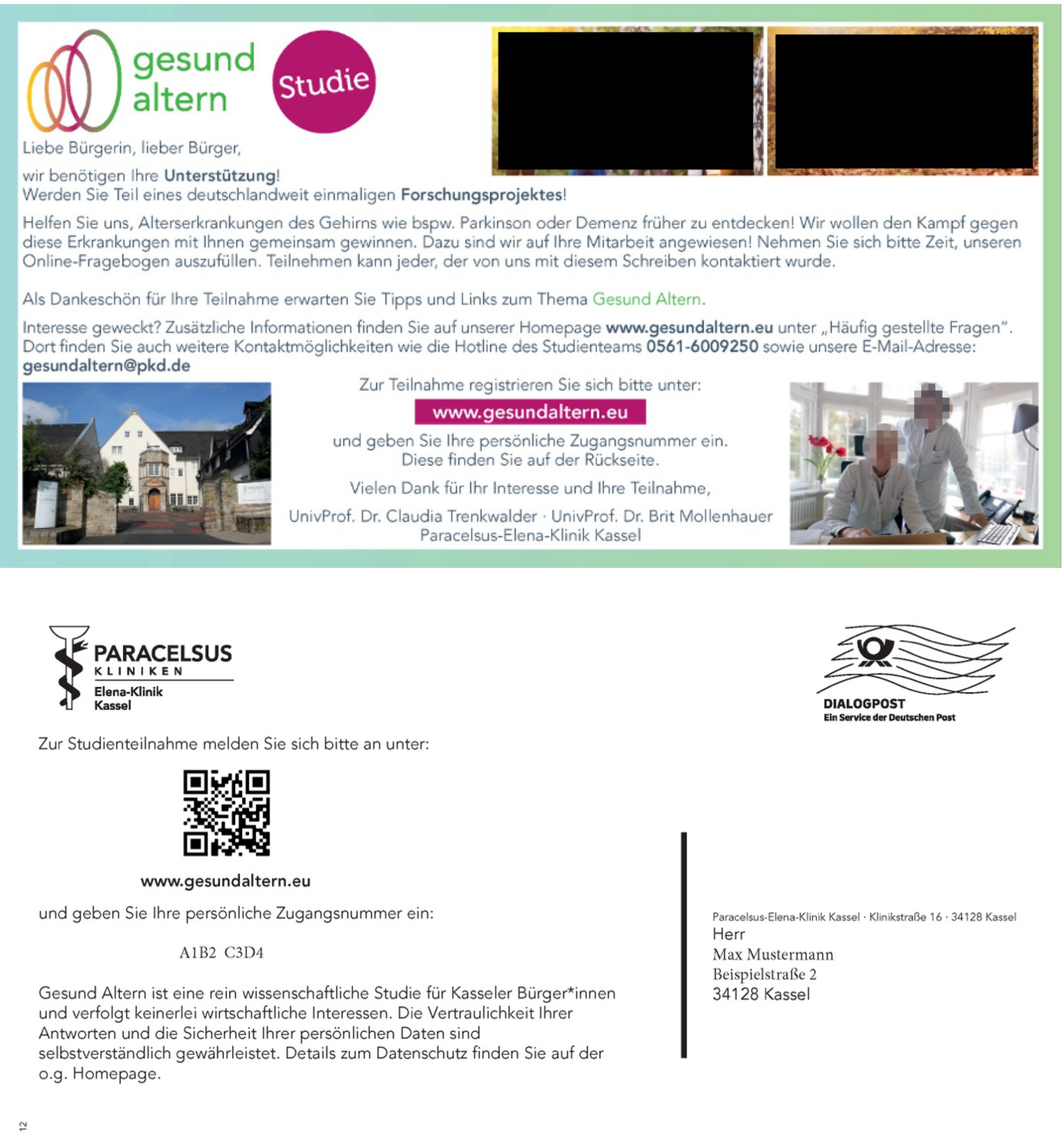

Supplement Figure 2 **Sample of the invitation card**. The front (upper half) is generic and includes the invitation to take part in the online study as well as contact information. The back (lower part) is individualized with the address of the participant and a unique participation code (token). Photos under copyright were blackened out.
